# Associations of Cognitive Function Scores with Carbon Dioxide, Ventilation, and Volatile Organic Compound Exposures in Office Workers: A Controlled Exposure Study of Green and Conventional Office Environments

**DOI:** 10.1289/ehp.1510037

**Published:** 2015-10-26

**Authors:** Joseph G. Allen, Piers MacNaughton, Usha Satish, Suresh Santanam, Jose Vallarino, John D. Spengler

**Affiliations:** 1 Exposure, Epidemiology, and Risk Program, Department of Environmental Health, Harvard T.H. Chan School of Public Health, Boston, Massachusetts, USA; 2 Psychiatry and Behavioral Sciences, SUNY-Upstate Medical School, Syracuse, New York, USA; 3 Industrial Assessment Center, Center of Excellence, Syracuse University, Syracuse, New York, USA

## Abstract

**Background:**

The indoor built environment plays a critical role in our overall well-being because of both the amount of time we spend indoors (~90%) and the ability of buildings to positively or negatively influence our health. The advent of sustainable design or green building strategies reinvigorated questions regarding the specific factors in buildings that lead to optimized conditions for health and productivity.

**Objective:**

We simulated indoor environmental quality (IEQ) conditions in “Green” and “Conventional” buildings and evaluated the impacts on an objective measure of human performance: higher-order cognitive function.

**Methods:**

Twenty-four participants spent 6 full work days (0900–1700 hours) in an environmentally controlled office space, blinded to test conditions. On different days, they were exposed to IEQ conditions representative of Conventional [high concentrations of volatile organic compounds (VOCs)] and Green (low concentrations of VOCs) office buildings in the United States. Additional conditions simulated a Green building with a high outdoor air ventilation rate (labeled Green+) and artificially elevated carbon dioxide (CO2) levels independent of ventilation.

**Results:**

On average, cognitive scores were 61% higher on the Green building day and 101% higher on the two Green+ building days than on the Conventional building day (p < 0.0001). VOCs and CO2 were independently associated with cognitive scores.

**Conclusions:**

Cognitive function scores were significantly better under Green+ building conditions than in the Conventional building conditions for all nine functional domains. These findings have wide-ranging implications because this study was designed to reflect conditions that are commonly encountered every day in many indoor environments.

**Citation:**

Allen JG, MacNaughton P, Satish U, Santanam S, Vallarino J, Spengler JD. 2016. Associations of cognitive function scores with carbon dioxide, ventilation, and volatile organic compound exposures in office workers: a controlled exposure study of green and conventional office environments. Environ Health Perspect 124:805–812; http://dx.doi.org/10.1289/ehp.1510037

## Introduction

The increasing cost of energy in the 1970s led to a change in building practices throughout the United States as buildings were increasingly constructed to be airtight and energy efficient. These changes are reflected in decreasing air exchange rates in homes and office buildings. For homes, beginning in this time period, typical air exchange rates began decreasing from approximately 1 air change per hour (ACH) to approximately 0.5 ACH [Chan et al. 2003; Hodgson et al. 2000; American Society of Heating, Refrigerating, and Air-Conditioning Engineers (ASHRAE) 2013b].

Homes built since 2000 are designed to be even more energy efficient and therefore can be even tighter [0.1–0.2 ACH (Allen et al. 2012; ASHRAE 2013b)]. The > 100-year story of ventilation in buildings is complicated and was neatly summarized recently by Persily (2015). Persily describes the original ASHRAE 62 standard, issued in 1973, and the many subsequent iterations (e.g., ASHRAE 62.1 applies to commercial buildings), demonstrating the evolving nature of our understanding regarding the relationship between ventilation rate and acceptable indoor air quality. Similarly to the history of home ventilation, commercial ventilation requirements were lowered in the early 1980s, largely as an energy-conservation measure (Persily 2015).

With such design changes comes the potential for negative consequences to indoor environmental quality (IEQ) because decreased ventilation can lead to increased concentration of indoor pollutants. Building-related illnesses and sick building syndrome (SBS) were first reported in the 1980s as ventilation rates decreased (Riesenberg and Arehart-Treichel 1986), with significant annual costs and productivity losses due to health symptoms attributable to the indoor environment (Fisk and Rosenfeld 1997). A few factors of the indoor and work environments have been found to be associated with occupant health. These factors include environmental measures, such as humidity; building factors, such as ventilation rate; workspace factors, such as the presence of chemical-emitting materials; and personal factors, such as job stress, allergies, and sex (Mendell 1993; Wargocki et al. 2000; Bornehag et al. 2005; Hedge 2009; Hedge and Gaygen 2010; Nishihara et al. 2014).

The IEQ problems that arose from conventional buildings with a tight envelope contributed to the advent of sustainable design or “green” building rating systems [e.g., U.S. Green Building Council’s (USGBC’s) Leadership in Energy and Environmental Design (LEED®)]. These rating systems aim to reduce the environmental footprint of buildings and to improve occupant health by providing design credits to new and existing buildings for adopting green design, operation, and maintenance. Different levels of ratings for the building are then awarded based on the number of acquired credits (e.g., silver, gold, platinum) (USGBC 2014). Many design credits are aimed at energy efficiency and environmental performance but also include guidelines for improving ventilation and filtration, using low-emitting materials, controlling indoor chemical and pollutant sources, improving thermal and lighting conditions, and offering daylight views to building occupants (USGBC 2014). Compared with conventional buildings, environmental measurements in green buildings show lower concentrations of several key pollutants including particles, nitrogen dioxide, volatile organic compounds (VOCs), and allergens (Colton et al. 2014; Jacobs et al. 2015; Noris et al. 2013). However, these reductions generally did not extend to carbon dioxide (CO_2_) or air exchange rate, demonstrating the influence of energy efficiency on green building operation and design. Green buildings were associated with improved IEQ and have been associated with reductions in self-reported symptoms in people inhabiting the buildings and with improved productivity in home, school, and office settings (Colton et al. 2014; NRC 2007; Singh et al. 2010). However, an important limitation of these studies is their reliance on subjective outcome measures, such as surveys, that have the potential for bias because participants are aware of their status (i.e., green or control). To date, we know of no studies that have been conducted in green buildings where participants were blinded to their building condition (Allen et al. 2015).

We designed this study to objectively quantify the impact of indoor environment on higher-order cognitive function, a driver of real-world productivity in office workers. We simulated low-VOC (“Green”) and high-VOC (“Conventional”) building conditions, both at the ASHRAE standard ventilation rate. Recognizing that technological advances in mechanical systems open the possibility of increasing ventilation rates without sacrificing energy efficiency, we also tested another building condition that introduced higher rates of ventilation to the Green building condition. This condition was labeled Green+. Last, we were motivated by the recent findings by Satish et al. (2012) that CO_2_ may be a direct pollutant and not just an indicator of ventilation; therefore, we assessed cognitive function after a full-workday exposure to CO_2_ while holding other variables constant.

## Methods

### Study Design

This study was undertaken in a controlled office environment to estimate the effects of several indoor environmental quality parameters on an objective measure of cognitive function. We used a double-blinded study design that included repeated measures of cognitive function on the same individual, characterization of potential confounding IEQ variables, and midweek testing to avoid Monday/Friday effects. All participants received the same exposures on each day, with exposures varying each day.

### Study Population

Twenty-four professional-grade employees (architects, designers, programmers, engineers, creative marketing professionals, managers) in the Syracuse, New York, area participated in a 6-day longitudinal study of cognitive performance and building conditions (Table 1). Six additional people were originally recruited as backups but were not enrolled in the study. Participants were recruited through emails to local businesses. The study population was restricted to nonsensitive persons by excluding current smokers and people with asthma (because of testing indoor-air quality), claustrophobia, and schizophrenia (because this was an experiment where participants were required to remain in the laboratory). The participants were relocated to the Willis H. Carrier Total Indoor Environmental Quality (TIEQ) Laboratory at the Syracuse Center of Excellence (CoE) for 6 days over the course of 2 weeks in November of 2014. The study protocol was reviewed and approved by the Harvard T.H. Chan School of Public Health Institutional Review Board (IRB). SUNY Upstate Medical and Syracuse University ceded their review to Harvard’s IRB. All participants signed informed consent documents and were compensated with $800.

**Table 1:** Participant demographics.

Category	*n*	%
Sex
Male	10	42
Female	14	58
Age
20–30	8	33
31–40	3	12
41–50	6	25
51–60	4	17
61–70	3	12
Ethnicity
White/Caucasian	22	92
Black or African American	1	4
Latino	1	4
Highest level of schooling**
High school graduate	1	4
Some college	2	8
College degree	13	54
Graduate degree	8	33
Job category
Managerial	5	21
Professional	15	63
Technical	1	4
Secretarial or clerical	1	4
Other	2	8

Participants reported to the CoE on Tuesday, Wednesday, and Thursday, at 0900 hours, for 2 consecutive weeks. The CoE has two nearly identical office environments located adjacent to one another as part of the TIEQ Lab, each with 12 cubicles. The rooms are similarly constructed and have identical building materials (e.g., carpeting, cubicles, painting, computers). Environmental conditions, described in the following sections, were designed to be consistent in the two rooms. On the first day, the participants were randomly assigned to cubicles in the TIEQ Lab for the duration of the study. Participants were requested to spend the entire work day in the simulated office environments performing their normal work activities. They were provided with computers, internet access, and an area for private telephone calls and printing. A 45-min lunch break was given between 1200 and 1245 hours (Room 1) or 1215 and 1300 hours (Room 2). A limited selection of food was provided, served, and eaten in a room adjacent to the two simulated office environment rooms. Participants then returned to the simulated office environment to continue their work. Cognitive testing was initiated at 1500 hours each day, after which the participants completed the daily surveys and left the TIEQ Lab. Participants were blinded to test conditions, as were the analysts performing the cognitive function assessment. Participants were not given any instructions regarding how to spend their time in the evenings or on the Mondays before starting the test period.

### Indoor Environment Simulation

The different environmental simulations in the TIEQ Lab on each day were designed to evaluate commonly encountered conditions and guidance values (Table 2). The three test parameters that were experimentally controlled were ventilation with outdoor air, CO_2_, and VOCs. We selected two outdoor air ventilation rates for this study: 20 cfm/person and 40 cfm/person. LEED® specifies that mechanically ventilated spaces must meet ventilation rates under ASHRAE 62.1 or the local equivalent, whichever is more stringent (USGBC 2014; ASHRAE 2013a). Many local building codes use the previous ASHRAE standard of 20 cfm/person, which corresponds to an indoor CO_2_ concentration of 945 ppm. Therefore, 20 cfm/person was the ventilation rate we used for the Green and Conventional simulation days because it reflects the minimum required ventilation rate for both green buildings (through LEED®) and conventional buildings (through ASHRAE). We also sought to evaluate the impact of a doubling of that minimum rate to 40 cfm/person (labeled Green+ days), which corresponds to an approximate steady-state CO_2_ concentration of 550 ppm. To ensure blinding, air movement was maintained at 40 cfm per person on all study days, with 100% outdoor air ventilation used on Green+ days and moderate and high CO_2_ days, and a mix of 50% outdoor air and 50% recirculated air used on the Green and Conventional days to achieve 20 cfm outdoor air ventilation per person.

**Table 2:** Average indoor environmental conditions simulated in each room of the TIEQ lab.

Variable	Day 1 Green+		Day 2 Moderate CO_2_		Day 3 High CO_2_		Day 4 Green		Day 5 Conventional		Day 6 Green+	
Date	4 November		5 November		6 November		11 November		12 November		13 November	
Day of the week	Tuesday		Wednesday		Thursday		Tuesday		Wednesday		Thursday	
Room	502	503	502	503	502	503	502	503	502	503	502	503
Experimental parameters
CO_2_ (ppm)	563	609	906	962	1,400	1,420	761^*b*^	726^*b*^	969	921	486	488
Outdoor air ventilation (cfm/person)^*a*^	40	40	40	40	40	40	20	20	20	20	40	40
TVOCs (μg/m^3^)	43.4	38.5	38.2	28.6	32.2	29.8	48.5	43.5	506	666	55.8	14.9
Other environmental parameters
Temperature (°C)	23.9	24.5	22.4	23.9	21.3	22.0	22.9	23.7	21.8	22.5	20.7	21.3
Relative humidity (%)	31.0	30.4	34.2	31.6	38.7	38.3	34.3	33.3	39.6	38.3	27.8	26.8
NO_2_ (μg/m^3^)	57.9	58.9	53.2	54.1	60.8	58.4	51.3	45.6	54.6	50.8	56.5	55.5
O_3_ (μg/m^3^)	3.42	21.2	14.4	13.0	1.37	0.00	6.85	238	1.71	1.37	4.11	6.85
PM_2.5_ (μg/m^3^)	2.38	3.49	3.35	2.58	2.97	2.42	1.26	1.83	1.68	1.34	1.26	1.38
Noise (dB)	51.3	49.9	49.7	48.8	52.5	48.8	49.6	48.7	51.1	48.8	50.5	49.2
Illuminance (mV)	2.95	2.70	2.89	2.83	2.31	2.04	3.11	2.93	2.74	2.51	2.39	2.28
Irradiance (mV)	9.07	8.76	9.45	9.37	6.00	6.05	9.90	9.60	8.30	8.14	6.70	6.82
Abbreviations: TIEQ, Total Indoor Environmental Quality; TVOCs, total volatile organic compounds. ^***a***^A constant air flow rate of 40 cfm/person was maintained on all study days, with 100% outdoor air used on days 1, 2, 3, and 6 and 50% outdoor air and 50% recirculated air used to achieve an outdoor air ventilation rate of 20 cfm/person on days 4 and 5. ^***b***^Average concentration from 1400 to 1700 hours was 926 ppm, but lower CO_2_ concentrations in the morning hours during the approach to steady state led to a lower average CO_2_ concentration.												

For the assessment of the independent association of CO_2_ concentration with cognitive function, the outdoor air ventilation rate was held constant at 40 cfm/person while CO_2_ was added to the chambers to reach three steady-state CO_2_ concentrations. The first target was 550 ppm (Green+, Days 1 and 6). The second target, 945 ppm, was selected to reflect a level that would be expected at the previously described ASHRAE minimum recommended ventilation rate of 20 cfm outdoor air/person. The third target, 1,400 ppm, was selected to represent a higher, but not uncommon, concentration of CO_2_ found in indoor environments [1,400 ppm is the maximum observed 8-hr time-weighted-average CO_2_ concentration in the U.S. Environmental Protection Agency (EPA) BASE data set (U.S. EPA 1998)]. On Days 2 and 3, when the independent effects of CO_2_ were tested, CO_2_ was added from a cylinder of ultra-pure CO_2_ (≥ 99.9999% pure) to the TIEQ Lab supply air at a rate needed to maintain steady-state CO_2_ concentrations of 945 ppm and 1,400 ppm. Because CO_2_ concentrations are affected by occupancy and mixing impact concentrations, a technician monitored CO_2_ in real time and adjusted the emission rate accordingly to maintain constant CO_2_ concentrations. During Days 4 and 5 (Green and Conventional), injection of pure CO_2_ was not needed to reach the target CO_2_ concentrations because of the reduced outdoor ventilation rate. A protocol was established to ensure participant safety in the event that there were unexpected deviations. CO_2_ was monitored in real time at a high spatial resolution in the test rooms using three different and independently calibrated monitors. A technician seated next to the CO_2_ shut-off valves monitored the CO_2_ concentrations during the entire test period. The protocol called for immediately canceling the testing if CO_2_ concentrations exceeded preset thresholds that were well below occupational health limits [2,500 ppm, one-half of the threshold limit value set by the American Conference of Governmental Industrial Hygienists (ACGIH 2015)]. No deviations from protocol occurred during the study.

The TIEQ Lab was constructed with low-VOC materials, and low levels of VOCs were confirmed by pretesting (Table 3). To simulate a conventional office space with elevated VOCs, we placed VOC sources in the diffuser that supplied air to each cubicle area before the participants arrived on Day 5. We selected a target total VOC (TVOC) level of 500 μg/m^3^ based on the LEED® Indoor Air Quality Assessment credit limit, as measured using U.S. EPA method TO-15 (USGBC 2014). The diffusers were built into the floor of the TIEQ Lab, and there were no visible indicators of these sources for the participants to observe. We selected a mix of nonodoriferous sources to simulate VOC-emitting materials that are commonly found in office buildings and that covered four indoor VOC source categories including building materials [56 in^2^ (360 cm^2^) exposed edge melamine, 56 in^2^ (360 cm^2^) exposed edge particle board, 64 in^2^ (415 cm^2^) vinyl mat], adhesives [80 in^2^ (520 cm^2^) duct tape, 80 in^2^ (520 cm^2^) packing tape (exposed)], cleaning products [1 oz. (30 mL) multi-surface cleaner, 4 multi-surface wipes, 144 in^2^ (930 cm^2^) recently dry-cleaned cloth], and office supplies (4 dry erase markers, 1 open bottle of correction fluid).

**Table 3:** Speciated VOC concentrations (μg/m^3^) on each study day, averaged across rooms.

Analyte	Condition						
	Background	Day 1 Green+	Day 2 Med. CO_2_	Day 3 High CO_2_	Day 4 Green	Day 5 Conventional	Day 6 Green+
VOCs
1,2,4-Trimethylbenzene	0.3	0.2	ND	0.1	ND	0.5	0.1
2-Butanone	2.5	0.7	0.7	0.8	1.1	1.1	0.6
2-Propanol	1.0	1.2	1.1	3.1	1.2	312.5	8.2
Acetone	12.0	14.7	9.6	8.7	20.0	20.0	8.6
Benzene	0.5	0.8	0.5	0.9	0.7	0.5	0.5
Carbon disulfide	0.6	0.2	ND	ND	ND	ND	0.1
Carbon tetrachloride	ND	0.2	0.4	ND	0.2	ND	ND
Chloroform	ND	0.1	ND	ND	ND	0.1	ND
Chloromethane	1.3	1.7	1.5	1.4	1.9	1.5	1.4
Cyclohexane	0.2	0.3	0.4	0.5	0.1	0.4	0.3
Dichlorodifluoromethane	2.5	2.6	2.9	2.7	2.9	2.4	2.5
Ethyl acetate	ND	ND	ND	ND	1.0	2.0	ND
Ethylbenzene	0.3	0.4	ND	0.3	0.2	0.1	0.1
Freon 113	0.3	0.7	0.8	0.8	0.8	0.2	0.4
Heptane	ND	0.3	ND	0.3	ND	257.5	6.9
Hexane	0.4	0.7	0.5	0.7	0.4	0.8	1.3
*m*,*p*-Xylene	0.8	1.5	0.4	1.0	1.0	0.7	0.7
Methylene chloride	0.5	0.3	0.6	0.5	0.3	0.4	0.4
*o*-Xylene	0.3	0.4	ND	0.4	0.1	0.3	0.1
Styrene	0.1	ND	ND	ND	ND	ND	0.1
Tetrachloroethene	3.7	0.9	ND	ND	0.9	0.6	0.2
Tetrahydrofuran	ND	ND	ND	ND	0.2	0.1	0.2
Toluene	2.4	2.1	1.4	1.9	2.2	1.9	2.9
*trans*-1,2-Dichloroethene	19.0	8.8	12.6	6.2	10.3	21.8	8.7
Trichloroethene	ND	ND	ND	ND	ND	ND	0.2
Trichlorofluoromethane	1.3	1.2	1.6	1.4	1.5	1.1	1.2
Grand total	50.0	40.1	35.0	31.4	46.9	626.4	45.6
Aldehydes
2,5-Dimethylbenzaldehyde	ND	ND	ND	ND	ND	ND	ND
Acetaldehyde	1.0	3.7	3.2	3.1	5.4	7.3	2.1
Benzaldehyde	ND	ND	ND	ND	ND	1.5	ND
Crotonaldehyde	ND	ND	ND	ND	ND	ND	ND
Formaldehyde	2.4	5.9	5.5	5.4	8.9	11.7	4.4
Hexanaldehyde	ND	0.8	0.8	ND	1.9	2.4	ND
Isovaleraldehyde	ND	ND	ND	ND	ND	ND	ND
*m*,*p*-Tolualdehyde	ND	ND	ND	ND	ND	ND	ND
*n*-Butyraldehyde	1.1	2.7	1.4	2.3	2.8	2.4	2.0
*o*-Tolualdehyde	ND	ND	ND	ND	ND	ND	ND
Propionaldehyde	ND	0.7	1.2	ND	1.4	1.6	0.6
Valeraldehyde	ND	ND	ND	ND	ND	ND	ND
Glutaraldehyde	ND	0.5	ND	ND	0.4	ND	ND
*o*-Phthalaldehyde	ND	65.1	57.7	70.0	41.6	38.4	76.8
Grand total	4.6	79.4	69.8	80.9	62.4	65.3	85.8
Abbreviations: ND, non-detect; VOC, volatile organic compound.							

### Environmental Monitoring

The study team characterized the TIEQ Lab on each test day for a wide range of IEQ indicators: CO_2_, temperature, relative humidity, barometric pressure, sound levels, VOCs, aldehydes, nitrogen dioxide (NO_2_), ozone (O_3_), particulate matter ≤ 2.5 μm in diameter (PM_2.5_), and light. Netatmo Weather Stations were installed in each cubicle to measure temperature, humidity, carbon dioxide concentrations (parts per million), and sound levels (decibels) every 5 min for each participant. The stations were calibrated to 0 and 3,000 ppm CO_2_ using calibration gases and were validated using a calibrated TSI Q-Trak (model 7575). In addition, the Netatmos were tested with 400 and 1,000 ppm calibration gas at the end of the study to determine if the sensors drifted during the 2-week period. Duplicate measures of CO_2_ were collected in each room using a TSI Q-Trak model 7575 and two K-33 data loggers. Summa canisters were used to detect overall levels of 62 common VOCs in a randomly selected workstation in each room for each of the study days (Table 3). An additional sample was collected in a third randomly selected cubicle each day. Samples were analyzed by ALS Laboratories according to U.S. EPA method TO-15 (U.S. EPA 1999). Thirty-six VOCs were not detected in any of the samples.

In each room, a monitoring station was placed at the far end of the room from the entrance to monitor additional IEQ parameters. The station included *a*) a TSI SidePak AM510 personal aerosol monitor to measure PM_2.5_, *b*) an integrated filter sample for gravimetric analysis of PM_2.5_ and elemental composition, *c*) an 8-hr integrated active air sample (0.4 L/min flow rate) analyzed for 14 aldehydes by ALS Analytical Laboratories using U.S. EPA method TO-11 (U.S. EPA 1999), *d*) a passive NO_2_ badge [8-hr time-weighted average; model X-595, Assay Technology; Occupational Safety and Health Administration (OSHA) method 182 (OSHA 1991)], *e*) a passive sampling badge for O_3_ [8-hr time-weighted average; model X-586, Assay Technology; OSHA Method 214 (OSHA 2008)], and *f*) illuminance and irradiance measures using an IL1400 radiometer/powermeter with SEL-033/Y/W and SEL-033/F/W detectors. VOC, aldehyde, NO_2_, O_3_, and integrated PM_2.5_ samples had at least one blank and one duplicate for every 10 samples. Samples were blank-corrected for analyses. All duplicate measures were within 15% of each other, and an average of the two was used for subsequent analyses.

An ambient air monitoring system was installed on the roof of the CoE to measure PM_2.5_, O_3_, and NO_2_ using the same procedures and equipment as the indoor stations to establish the potential influence of outdoor contaminants on the indoor environment. Outdoor temperature, humidity, solar radiation, and wind speed/direction data were obtained from the CoE weather station located on the roof of the building. Baseline (i.e., before occupancy) measurements of all IEQ parameters were collected in the TIEQ Lab 1 month before the study was performed.

### Cognitive Function Assessment

The cognitive assessment was performed daily using the Strategic Management Simulation (SMS) software tool, which is a validated, computer-based test that has been designed to test the effectiveness of management-level employees through assessments of higher-order decision making (Streufert et al. 1988; Breuer and Satish 2003; Satish et al. 2004). At the start of the 1.5-hr test, participants were given a brief, 1-page description of the scenario that they were about to participate in during the test. They were then logged onto a standardized desktop computer station at the TIEQ Lab using a unique identifier. Participants were not allowed to use their own computers and were instructed to turn off all other devices before the assessment. The simulation was then initiated. Participants were exposed to diverse situations based on real-world equivalent challenges (e.g., handling a township in the role of a mayor or emergency coordinator). These scenarios are designed to capture participants’ standard response pattern. The software allows flexibility in approach; participants can choose to make a decision or form a plan at any time in response to any stimulus from the program. The absence of requirements or stated demands allows the participant the freedom to strategize and take initiative in his or her typical cognitive style. Based on the participant’s actions, plans, responses to incoming information, and use of prior actions and outcomes, the SMS software computes scores for nine cognitive factors (Table 4).

**Table 4:** Description of the cognitive domains tested.

Cognitive function domain^*a*^	Description
Basic Activity Level	Overall ability to make decisions at all times
Applied Activity Level	Capacity to make decisions that are geared toward overall goals
Focused Activity Level	Capacity to pay attention to situations at hand
Task Orientation	Capacity to make specific decisions that are geared toward completion of tasks at hand
Crisis Response	Ability to plan, stay prepared, and strategize under emergency conditions
Information Seeking	Capacity to gather information as required from different available sources
Information Usage	Capacity to use both provided information and information that has been gathered toward attaining overall goals
Breadth of Approach	Capacity to make decisions along multiple dimensions and use a variety of options and opportunities to attain goals
Strategy	Complex thinking parameter that reflects the ability to use well-integrated solutions with the help of optimal use of information and planning
^***a***^See Streufert et al. (1986) for detailed descriptions.	

A technician trained in administering this test was present to provide standardized instructions and periodically answer any questions from participants. Parallel scenarios (i.e., equivalent scenarios) were used from one day to the next, which allows individuals to be retested without potential bias caused by experience and learning effects (Swezey et al. 1998). Parallel scenarios have correlation coefficients between 0.68 and 0.94 for the scores on these cognitive function domains (Streufert et al. 1988).

### Statistical Analyses

**Figure 1:**
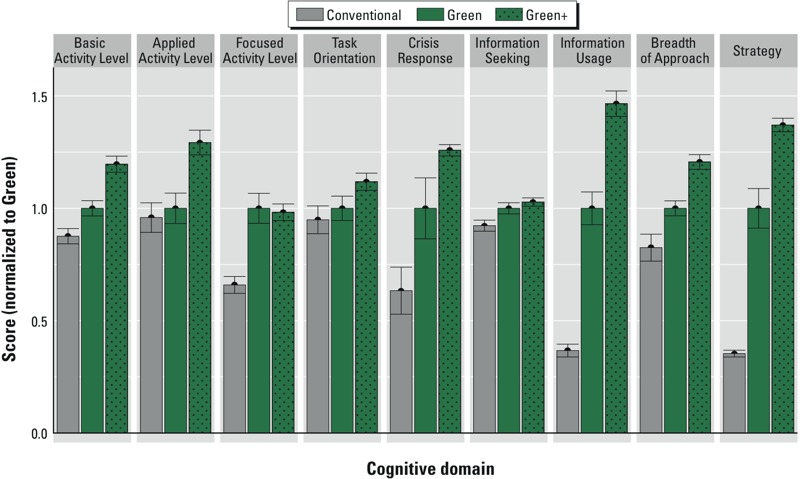
Average cognitive function scores and standard error bars by domain for the Conventional, Green, and two Green+ conditions, normalized to the Green condition by dividing all scores by the average score during the Green condition.

**Figure 2:**
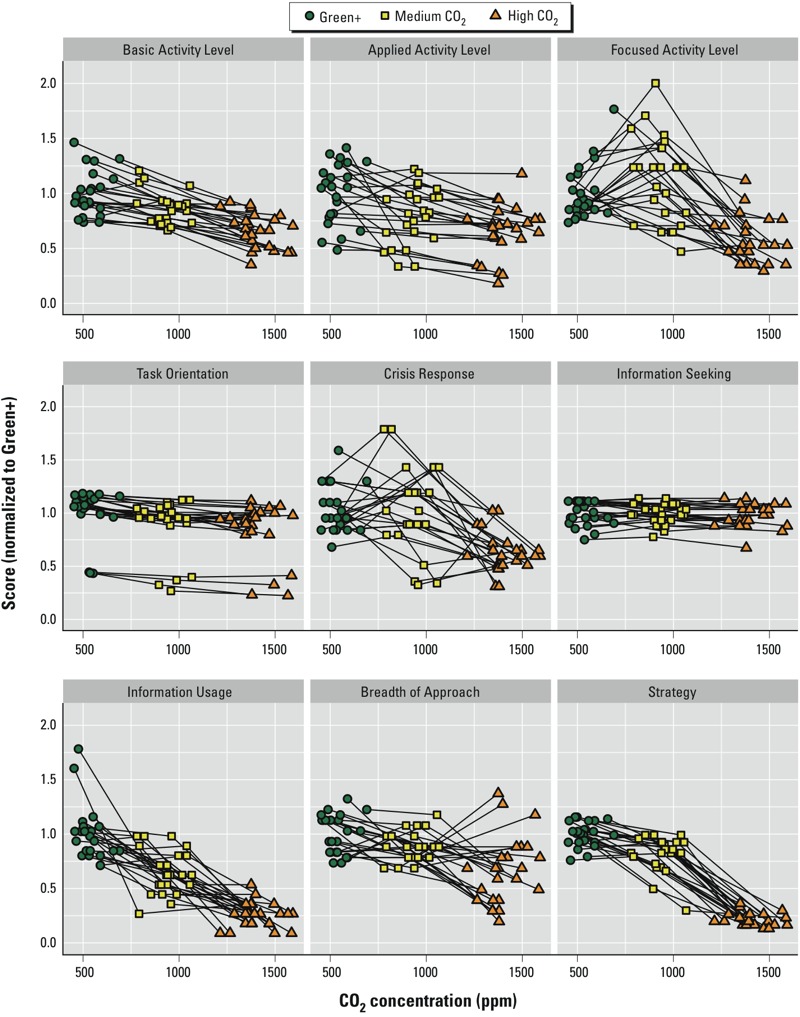
Cognitive function scores by domain and participant and the corresponding carbon dioxide concentration in their cubicles. Each line represents the change in an individual’s CO_2_ exposure and cognitive scores from one condition to the next, normalized to the average CO_2_ exposure across all participants during the Green+ conditions.

Generalized additive mixed effect models were used to test associations between environmental exposures and cognitive function while controlling for the correlated nature of the repeat measures. In the model, the most specific exposure was assigned to each participant, whether it be cubicle-level (CO_2_), room-level (VOCs), or lab-level (ventilation). Participant ID was treated as a random intercept to control for confounding by individual characteristics. The residuals were normally distributed and homoscedastic for all models (data not shown). We used penalized splines to graphically assess linearity in the associations between environmental exposures and cognitive scores. SMS scores are often compared with normative data from other uses of the SMS software (e.g., Satish et al. 2012). Because we did not have access to normative data, we instead used our study population as the reference group. Based on the analysis, cognitive scores were normalized to Conventional (Table 5), Green (Figure 1), or Green+ (Figure 2) scores to allow for comparisons across cognitive function domains, each of which has a unique scale in its raw form. The scores were normalized for each cognitive domain by dividing all scores by the average score obtained during the normalizing condition. The statistical significance of our results was not affected by normalization. Given the multiple comparisons tested in this analysis, *p*-values < 0.001 were considered to be statistically significant after performing a Bonferroni correction. Analyses were performed using the open-source statistical package R v.3.0.0 (R Core Team 2015).

**Table 5:** Generalized additive mixed effect models testing the effect of IEQ condition and on cognitive scores, normalized to the “Conventional” condition, treating participant as a random intercept.

Condition	Cognitive domain: estimate, [95% confidence interval], (*p*-value)									
	Basic Activity Level	Applied Activity Level	Focused Activity Level	Task Orientation	Crisis Response	Information Seeking	Information Usage	Breadth of Approach	Strategy	Average
Day 1 Green+	1.35 [1.28, 1.43] (< 0.0001)	1.39 [1.26, 1.52] (< 0.0001)	1.44 [1.27, 1.62] (< 0.0001)	1.14 [1.11, 1.17] (< 0.0001)	2.35 [1.91, 2.78] (< 0.0001)	1.10 [1.07, 1.14] (< 0.0001)	3.94 [3.47, 4.41] (< 0.0001)	1.43 [1.25, 1.60] (< 0.0001)	3.77 [3.40, 4.14] (< 0.0001)	1.99 [1.89, 2.09] (< 0.0001)
Day 2 Moderate CO_2_	1.20 [1.13, 1.27] (< 0.0001)	1.08 [0.95, 1.21] (0.23)	1.68 [1.51, 1.85] (< 0.0001)	1.05 [1.02, 1.08] (0.0009)	2.05 [1.63, 2.48] (< 0.0001)	1.11 [1.08, 1.15] (< 0.0001)	2.61 [2.15, 3.07] (< 0.0001)	1.29 [1.12, 1.46] (0.0013)	3.17 [2.81, 3.53] (< 0.0001)	1.69 [1.59, 1.79] (< 0.0001)
Day 3 High CO_2_	0.91 [0.84, 0.98] (0.015)	0.88 [0.75, 1.01] (0.081)	0.85 [0.68, 1.02] (0.087)	1.00 [0.97, 1.03] (0.76)	1.33 [0.90, 1.75] (0.14)	1.08 [1.05, 1.12] (< 0.0001)	1.01 [0.55, 1.48] (0.95)	0.98 [0.81, 1.15] (0.78)	0.83 [0.47, 1.19] (0.36)	0.99 [0.89, 1.09] (0.78)
Day 4 Green	1.14 [1.06, 1.21] (0.0003)	1.04 [0.91, 1.18] (0.51)	1.51 [1.34, 1.68] (< 0.0001)	1.03 [1.00, 1.06] (0.065)	1.97 [1.54, 2.40] (< 0.0001)	1.09 [1.05, 1.12] (< 0.0001)	2.72 [2.26, 3.19] (< 0.0001)	1.21 [1.04, 1.38] (0.018)	2.83 [2.46, 3.19] (< 0.0001)	1.61 [1.51, 1.71] (< 0.0001)
Day 5 Conventional (Reference)	1.00	1.00	1.00	1.00	1.00	1.00	1.00	1.00	1.00	1.00
Day 6 Green+	1.37 [1.30, 1.44] (< 0.0001)	1.33 [1.20, 1.46] (< 0.0001)	1.52 [1.35, 1.69] (< 0.0001)	1.15 [1.12, 1.19] (< 0.0001)	2.27 [1.85, 2.69] (< 0.0001)	1.11 [1.08, 1.15] (< 0.0001)	4.04 [3.58, 4.51] (< 0.0001)	1.50 [1.33, 1.67] (< 0.0001)	3.98 [3.62, 4.34] (< 0.0001)	2.03 [1.93, 2.13] (< 0.0001)
*R*^2^	0.34	0.17	0.33	0.03	0.28	0.06	0.69	0.27	0.79	0.81
IEQ, indoor environmental quality.										

## Results

### Green Building and Cognitive Function

The TVOC levels were constant at < 50 μg/m^3^ on all study days except the Conventional building day, when levels increased to 506–666 μg/m^3^ depending on the room. The compounds that increased in concentration included but were not limited to formaldehyde, benzaldehyde, acetaldehyde, heptane, and 2-propanol. Heptane and 2-propanol had the largest increases of the sampled compounds (Table 3). Total aldehyde concentrations were primarily driven by *o*-phthalaldehyde and remained relatively constant on all study days.

Cognitive function scores were higher under the Green building condition than under the Conventional building condition for all nine functional domains (Figure 1). On average, cognitive scores were 61% higher on the Green building day and 101% higher on the two Green+ building days than on the Conventional building day. The largest effects were seen for Crisis Response, Information Usage, and Strategy, all of which are indicators of higher-level cognitive function and decision making (Streufert and Swezey 1986). For Crisis Response, scores were 97% higher during the Green condition than during the Conventional condition, and 131% higher during the Green+ condition than during the Conventional condition. For Information Usage, scores obtained under the Green and Green+ conditions were 172% and 299% higher than under the Conventional condition, respectively. Finally, for Strategy, which tested the participants’ ability to plan, prioritize, and sequence actions, the Green and Green+ day scores were 183% and 288% higher than on the Conventional day, respectively (Table 5).

The raw cognitive scores for each domain were normalized to the Conventional condition and modeled by study day, controlling for participant (Table 5). The repeat simulation of the Green+ day (Day 6), which was added to the study as a quality control measure, showed similar cognitive function scores: *p*-values for the null hypothesis of no difference between the 2 days ranged from 0.27 for Strategy (normalized scores of 3.77 and 3.98, respectively) to 0.73 for Crisis Response (normalized scores of 2.35 and 2.27). Under the Green+ condition, participants had statistically significantly higher cognitive function scores than under the Conventional condition in all domains (*p* < 0.0001). Under the Green condition, particpants had higher scores than under the Conventional condition in all domains, five of which were statistically significant.

Participants scored higher on the Green+ days than on the Green day in eight of nine domains, resulting in a 25% increase in scores on average when outdoor air ventilation rates were increased. Cognitive scores were 20% higher on the Green+ days than on the moderate CO_2_ day when CO_2_ levels were higher (*p*-value < 0.0001), and 5% higher on the moderate CO_2_ day than on the Green day when outdoor air ventilation was reduced (*p*-value = 0.12). These estimates and *p*-values were produced by rerunning the “average” model in Table 5 with the Green condition as the reference category (data not shown).

The model of the average scores in Table 5 had a high *R* ^2^ value of 0.81, indicating that a significant amount of the variability in cognitive scores can be explained by these indoor-environment test conditions, leaving only 19% of the variability to be explained by all other potential intrapersonal drivers of cognitive function such as diet, the previous night’s sleep quality, and mood. For the specific domains of cognitive function, the *R* ^2^ ranged from 0.03 to 0.79.

### Carbon Dioxide and Cognitive Function

The effects of CO_2_ on cognitive function scores while all other parameters were held constant are depicted in Figure 2. Because the air in each room was not completely mixed, there was some variability in CO_2_ levels between cubicles. Each line represents the change in an individual’s CO_2_ exposure and cognitive scores from one condition to the next, normalized to the average CO_2_ exposure across all participants during the Green+ conditions. For seven of the nine cognitive function domains, average cognitive scores decreased at each higher level of CO_2_ (Table 5). Cognitive function scores were 15% lower for the moderate CO_2_ day (~ 945 ppm) and 50% lower on the day with CO_2_ concentrations of ~1,400 ppm than on the two Green+ days (Table 5, dividing the average Green+ estimate by the moderate CO_2_ and high CO_2_ estimates, respectively). The exposure–response curve between CO_2_ and cognitive function is approximately linear across the CO_2_ concentrations used in this study; however, whether the largest difference in scores is between the Green+ conditions and the moderate CO_2_ condition or the moderate CO_2_ condition and the high CO_2_ condition depends on the domain (Figure 2).

Ventilation rate, CO_2_, and TVOCs were modeled separately from study day to capture the independent effects of each factor on cognitive function scores, averaged across all domains. A statistically significant increase in scores was associated with ventilation rate, CO_2_, and TVOCs (*p* < 0.0001 for all three parameters). On average, a 400-ppm increase in CO_2_ was associated with a 21% decrease in a typical participant’s cognitive scores across all domains after adjusting for participant (data not shown), a 20-cfm increase in outdoor air per person was associated with an 18% increase in these scores, and a 500-μg/m^3^ increase in TVOCs was associated with a 13% decrease in these scores. Although other environmental variables were not experimentally modified, some did vary over the course of the study (Table 2). There was a high degree of consistency in IEQ between the two rooms; however, ozone was significantly higher in one of the chambers on the Green day. Cognitive scores were 4% higher in the room with high ozone on this day, after accounting for baseline cognitive performance in the two rooms. These IEQ parameters were added to the model with the experimentally controlled variables and were not found to be significantly associated with cognitive function at the 0.05 significance level.

## Discussion

### Green Buildings and Health

We found that when participants spent a full day in a Green building, there was a significant increase in their cognitive function scores compared with when they spent a day in an environment that had been designed to simulate a conventional building by elevating VOC concentrations. The study was designed to represent conditions typically observed in many buildings; we did not include extreme exposures or choose uncommon VOC sources. Further, we selected our target levels of VOCs, ventilation rates and CO_2_ to be above and below the standards in LEED®, ASHRAE, and the U.S. EPA BASE study in order to evaluate how these common standards and guidelines perform (USGBC 2014, ASHRAE 2013a, U.S. EPA 1998). Our findings indicate that there may be benefits to meeting the LEED® VOC guideline of 500 μg/m^3^ and enhancing ventilation rates beyond the minimum requirement under ASHRAE.

The “Conventional” building simulation parameters in our study were based on conditions described in the U.S. EPA BASE study, which plausibly represent the upper end of performance for “typical” buildings in the United States in the 1990s because the owners were willing to participate in the study, introducing potential self-selection bias, and larger, “non-problem” buildings were preferentially recruited (Persily and Gorfain 2004). Therefore, the extent to which BASE buildings represent typical conventional buildings is unknown. Our findings show impacts above the 95th percentile of CO_2_ (945 ppm) and the mean VOC concentration in the BASE study (450 μg/m^3^); however, a larger proportion of the buildings in the BASE study would likely have exceeded these targets if “problem” buildings had been included in the recruitment process.

The VOC levels on the Conventional and Green/Green+ days straddled both the LEED® TVOC guidance concentration of 500 μg/m^3^ and the BASE mean concentration of 450 μg/m^3^. The common VOC sources that were added to the rooms during the Conventional building day led to increases in a range of VOCs. Previous testing with the SMS tool showed that 2 hr of painting, which exposed participants to VOCs, was associated with reductions in three of the five domains investigated (Satish et al. 2013). The lower TVOC concentrations (yet larger number of sources) in the present study were associated with statistically significant decrements in decision-making performance in five of the nine domains.

### Carbon Dioxide and Ventilation

Carbon dioxide concentration in indoor environments has long been used as an indicator of ventilation and as a proxy for indoor air quality (ASHRAE 2013b). However, this conventional thinking is being challenged as the evidence mounts for CO_2_ as a direct pollutant, not just a marker for other pollutants (Satish et al. 2012). We found statistically significant declines in cognitive function scores when CO_2_ concentrations were increased to levels that are common in indoor spaces (approximately 950 ppm). In fact, this level of CO_2_ is considered acceptable because it would satisfy ASHRAE’s ventilation rate guidance for acceptable indoor air quality. Larger differences were seen when CO_2_ was raised to 1,400 ppm.

Satish et al. used the SMS tool to test the effects of CO_2_ exposures on the cognitive function of 22 participants, using a controlled chamber and injection of ultra-pure CO_2_ (Satish et al. 2012). The authors reported effects on seven of nine cognitive function domains with increasing CO_2_ concentration. The SMS tool was also used to test the relationship between ventilation rate and cognitive function among 16 participants (Maddalena et al. 2015). Participants scored significantly lower on eight of nine domains at low ventilation rates (12.5 cfm of outdoor air/person). In contrast to the present study, these other studies had *a*) a single experimental parameter; *b*) half-day or shorter exposures; *c*) multiple experimental conditions per day; *d*) atypical exposure targets (2,500 ppm of CO_2_ and 12.5 cfm outdoor air/person); and *e*) primarily students and college-age adults. Despite these differences, our study found similar changes in cognitive scores from a unit change in CO_2_ or outdoor air ventilation. Associations were consistent *a*) in all three study populations, indicating that knowledge workers and students were equally affected by CO_2_ and outdoor air ventilation, and *b*) at different exposure durations, indicating that even short exposures are associated with cognitive function. Given the similarities in findings, there may not be a desensitization or compensatory response from prolonged exposure. More research is necessary to investigate the presence of these responses or the lack thereof.

The CO_2_ exposure levels used in this study are comparable to those in a variety of indoor locations. Assessment of public housing units in Boston found median CO_2_ levels to be 809 ppm in conventional apartments and 1,204 ppm in the newly constructed LEED® platinum apartments (Colton et al. 2014). Corsi et al. (2002) reported CO_2_ concentrations > 1,000 ppm in 66% of 120 classrooms in Texas, and Shendell et al. (2004) measured CO_2_ concentrations > 1,000 ppm in 45% of 435 classrooms in Washington and Idaho and reported that elevated CO_2_ concentrations were associated with increases in student absences.

### Strengths and Limitations

The study design has several notable strengths. These strengths include repeated measures of cognitive function on the same individual for control of between-subject variability, characterization of the TIEQ Lab for potential environmental confounders, repeated testing of the same condition 9 days apart on different days of the week, mid-week testing to avoid potential Monday/Friday bias, participants and cognitive function analysts blinded to test conditions, and the use of an objective measure of cognitive function.

The SMS tool is an objective assessment tool, unlike self-reported metrics, and thus is less susceptible to the participant’s environmental perceptions. Extensive work has been dedicated to testing the validity of the SMS software; correlations between scores on these tests and other measures of productivity such as income at age and job level at age exceed 0.6 (Streufert et al. 1988). The correlations are stronger for the more strategic domains, such as strategy, information usage, and crisis response, than for domains pertaining to activity, such as information search and activity level. The domains that were most affected by the exposures in this study are the same domains that are most closely related to other measures of productivity (Streufert et al. 1988). Lastly, the concordance of the scores for the two Green+ conditions suggests that *a*) the study was internally valid, *b*) there were no learning effects associated with the test, and *c*) day of the week (Tuesday vs. Thursday) was not a potential confounding variable.

The potential for confounding or effect modification by parameters measured or otherwise was reduced by the use of the controlled environment and by repeated measures on each participant. By testing on subsequent days, it is possible that effects from one condition were reflected in the scores obtained on the next day. The environmental factors that were not experimentally modified exhibited some variability owing to changes in outdoor conditions and participant behavior. In particular, ozone levels fluctuated significantly between some IEQ conditions (Table 2). Environmental factors other than outdoor air ventilation, CO_2_, and VOCs were not statistically significant predictors of cognitive scores, but the possibility of uncontrolled confounding by these factors cannot be excluded. The environmental conditions on each of the study days met design criteria. On one day (Day 4), CO_2_ levels were lower in the morning than in the afternoon, which influenced the reported mean concentration. The CO_2_ levels on this day were similar to the moderate CO_2_ and Conventional conditions (Day 5) during the time leading up to and during the cognitive test (926 ppm from 1400 to 1700 hours). This study used a controlled environment to individually control certain contaminants. Assessments performed in actual office environments are important to confirm the findings in a noncontrolled setting.

## Conclusion

Office workers had significantly improved cognitive function scores when working in Green and Green+ environments compared with scores obtained when working in a Conventional environment. Exposure to CO_2_ and VOCs at levels found in conventional office buildings was associated with lower cognitive scores than those associated with levels of these compounds found in a Green building. Using low-emitting materials, which is common practice in Green buildings, reduces in-office VOC exposures. Increasing the supply of outdoor air lowers exposures to not only CO_2_ and VOCs but also to other indoor contaminants. Green building design that optimizes employee productivity and energy usage will require adopting energy-efficient systems and informed operating practices to maximize benefits to human health while minimizing energy consumption. This study was designed to reflect indoor office environments in which large numbers of people work every day. These exposures should be investigated in other indoor environments, such as homes, schools, and airplanes, where decrements in cognitive function and decision making could have significant impacts on productivity, learning, and safety.

**Editor’s Note:** In the Information Seeking column in Table 5, the *p*-values for Day 2 (Moderate CO_2_), Day 3 (High CO_2_), and Day 4 (Green) have been changed to < 0.0001 from 0.61, 0.35, and 0.45, respectively. In the Information Usage column in Table 5, the *p*-value for Day 3 (High CO_2_) has been changed from < 0.0001 to 0.95. The previous *p*-values were from a different reference category that was subsequently changed during the peer-review process. The new *p*-values are consistent with the current reference category, and the conclusions of the manuscript are unaffected by these changes.
